# Identification of core genes associated with prostate cancer progression and outcome via bioinformatics analysis in multiple databases

**DOI:** 10.7717/peerj.8786

**Published:** 2020-03-31

**Authors:** Yutao Wang, Jianfeng Wang, Kexin Yan, Jiaxing Lin, Zhenhua Zheng, Jianbin Bi

**Affiliations:** 1 Department of Urology, The First Hospital of China Medical University, Shenyang, China; 2 Department of Dermatology, The First Hospital of China Medical University, Shenyang, China

**Keywords:** Prostate cancer, Prognosis factor, Biomarkers, GEO, TCGA

## Abstract

**Abstract:**

The morbidity and mortality of prostate carcinoma has increased in recent years and has become the second most common ale malignant carcinoma worldwide. The interaction mechanisms between different genes and signaling pathways, however, are still unclear.

**Methods:**

Variation analysis of GSE38241, GSE69223, GSE46602 and GSE104749 were realized by GEO2R in Gene Expression Omnibus database. Function enrichment was analyzed by DAVID.6.8. Furthermore, the PPI network and the significant module were analyzed by Cytoscape, STRING and MCODE.GO. Pathway analysis showed that the 20 candidate genes were closely related to mitosis, cell division, cell cycle phases and the p53 signaling pathway. A total of six independent prognostic factors were identified in GSE21032 and TCGA PRAD. Oncomine database and The Human Protein Atlas were applied to explicit that six core genes were over expression in prostate cancer compared to normal prostate tissue in the process of transcriptional and translational. Finally, gene set enrichment were performed to identified the related pathway of core genes involved in prostate cancer.

**Result:**

Hierarchical clustering analysis revealed that these 20 core genes were mostly related to carcinogenesis and development. CKS2, TK1, MKI67, TOP2A, CCNB1 and RRM2 directly related to the recurrence and prognosis of prostate cancer. This result was verified by TCGA database and GSE21032.

**Conclusion:**

These core genes play a crucial role in tumor carcinogenesis, development, recurrence, metastasis and progression. Identifying these genes could help us to understand the molecular mechanisms and provide potential biomarkers for the diagnosis and treatment of prostate cancer.

## Introduction

Prostate cancer is the second most common male malignancy tumor in the world (Farhood et al., 2019). The morbidity and mortality of prostate cancer has surpassed bladder cancer and kidney cancer. It is now the most common tumor in the adult urology department in China (Mayer et al., 2001). There are several factors can increase the risk of prostate cancer (for example, family factors, bald, gonorrhea, smoking; Lian et al., 2015; Rao et al., 2015; Islami et al., 2014), and genetic predisposition is considered to be one of the important factors of the incidence of prostate cancer (Ju-Kun et al., 2016; Jansson et al., 2012; Hemminki, 2012). At present, 100 susceptibility loci were identified, the mechanism of prostate cancer is incomprehensible, although a large number of studies have been conducted on the development and recurrence of prostate cancer. Prostate-specific antigen (PSA) test has been used to assist the diagnosis of prostate cancer. Early PSA detection, however, could lead to over diagnosis and over treatment of prostate cancer. More recently, advances in high-throughput sequencing and screening techniques have enabled us to screen differently expressed genes at the same time. Therefore, this article aimed to identify the core protein coding genes related to the progression of cancer recurrence, metastasis and prognosis by bioinformatics analysis. As errors in an individual dataset are unavoidable, multiple datasets were analyzed. False positive results could be ignored by independent mRNA microarray analysis; false negative results have been ignored by multiple mRNA microarrays intersection analysis. Thus, four kinds of combinations were established between four datasets downloaded from Gene Expression Omnibus (GEO), differentially expressed genes (DEGs) were identified between cancer and non cancer tissues when the screening criteria met any three of the four datasets (Li et al., 2017). Afterwards we obtained six core differential expression genes which were verified in TCGA PRAD, GSE21032 and other datasets, besides these six core genes acted as independent prognostic factors and positively correlated with Gleason score (Katarzyna, Patrycja & Maciej, 2015). The six real core genes were thereby identified as potential biomarkers for prostate cancer.

## Materials and Methods

### Microarray data

Five prostate cancer microarray datasets were obtained from NCBI GEO (https://www.ncbi.nlm.nih.gov/geo/) (Edgar, Domrachev & Lash, 2002): GSE104749 (Shan et al., 2017), GSE38241 (Aryee et al., 2013), GSE69223 (Meller et al., 2016) and GSE46602 (Mortensen et al., 2015), GSE21032 (Taylor et al., 2010). The platform for GSE104749 was GPL570 Affymetrix Human Genome U133 Plus 2.0 Array which contained 4 PCa excision tissue and 4 noncancerous excision tissue samples. The platform for GSE38241 was GPL4133 Agilent-014850 Whole Human Genome microarray 4 × 44 K G4112F (Feature Number version) which contained 21 PCa excision tissue and 18 noncancerous excision tissue samples. The platform for GSE69223 was GPL 570 Affymetrix Human Genome U133 Plus 2.0 Array which contained 15 PCa excision tissue samples and 15 noncancerous excision tissue samples. The platform for GSE46602 was GPL570 Affymetrix Human Genome U133 Plus 2.0 Array which contained 36 excision tissue and 14 noncancerous tissue samples. The platform for GSE21032 was GPL5188 which contained 150 prostate excision tissue samples and 29 normal adjacent benign prostate excision tissue samples. Meanwhile we also downloaded clinical information and gene matrix from TCGA database (https://genome-cancer.ucsc.edu/) (Cancer Genome Atlas Research Network TCGA, 2015) which contained 52 normal prostate tissue samples and 495 prostate cancer samples.

### Identification of DEGs

The GEO2R (http://www.ncbi.nlm.nih.gov/geo/geo2r) was used to screen DEGs between prostate carcinoma samples and noncancerous samples GSE104749, GSE38241, GSE69223, GSE46602 datasets. GEO2R is a tool which allows users to obtain DEGs by comparing different groups. The DEGs are screened and sorted by significance. The genes with |log2FC| (fold change) ≥ 1 and *P*-value < 0.01 were considered to be DEGs.

### GO and pathway enrichment analysis

The Database for Annotation, Visualization and Integrated Discovery (DAVID, v6.8) is a function enrichment tool which supplies biological explanations of gene lists and proteomic studies from high-throughput sequencing (Huang et al., 2007). Higher-order functions of cells and organisms were derived from KEGG (http://www.genome.ad.jp/kegg/) databases (Kanehisa, 2002). The biological process, molecular function and cellular component were analyzed in GO (http://www.geneontology.org) (Ashburner et al., 2000). A *P* value of < 0.05 was the criterion for significance.

### Conduction of protein–protein interaction network

The direct and indirect interactions between proteins were established by STRING database (http://string-db.org/) (Franceschini et al., 2013). The PPI network was analyzed by STRING database with a criterion of combined score >0.4 considered to be a significant result. The biological network visualization of the protein interactions was revealed by the open source software Cytoscape 3.7.1 (Smoot et al., 2011). The MCODE in Cytoscape was used to the 20 core genes and significant module (Bandettini et al., 2012). Afterwards the PPI network and the significant module were established with the criteria (MCODE scores > 5, degree cutoff = 2, node score cutoff = 0.2, Max depth = 100 and *k*-score = 2).

### Hub genes selection and prognosis analysis

The candidate core genes were identified with degrees ≥20 of the most significant model in PPI network. The co-expression network of the candidate genes was established by the online platform cBioPortal (http://www.cbioportal.org) (Gao et al., 2013). The phenotype analysis of the core genes was performed by a heat map that was conducted by UCSC (http://genome-cancer.ucsc.edu) (Haeussler et al., 2019). Afterwards, to further screen out the independent prognosis factors of prostate cancer, GSE21032 log2 mRNA expression data and the clinical information were download from cBioPortal (http://cbio.mskcc.org/cancergenomics/prostate/data) and (https://www.cbioportal.org/study/clinicalData?id=prad_mskcc). We analyzed the correlation between candidate key genes and phenotype in the GSE21032 data performed by box plots, KM curve and ROC curve, *P* value < 0.05 was the criterion for significance. In this way, we identified the real core genes and verified their important value in the large sample TCGA.

### External data set evaluation and verification

The core protein coding genes were identified, the prognosis and clinical value were illustrated in the above study. Furtherly, the interaction between the core genes and metastasis state were analyzed by Oncomine (http://www.oncomine.com) (Vanaja et al., 2003; Grasso et al., 2012; Varambally et al., 2005). The Human Protein Atlas (HPA) (http://www.proteinatlas.org/) is an open source database to explore the human proteome. HPA combines various omics techniques to map all human proteins in cells, tissues and organs and the expression of core genes were evaluated on transcriptional and translational level. The analysis of variance was applied to show the correlativity between the expression of core genes and clinical stage to reveal the expression difference among different stages, the clinical data was from TCGA clinical information.

### Gene set enrichment analysis

In set TCGA, samples were divided into two groups based on the expression of core genes. To predict the function and effect of core genes, gene set enrichment (GSEA) (http://software.broadinstitute.org/gsea/index.jsp) (Subramanian et al., 2005) was applied to identified the gene set that expressed relatively with our core genes, and analyzed the pathway the gene set involved in. Pathway with *P* < 0.05 was considered to be significance. Afterwards, we identified the most significance pathway by taking the intersection of core genes. The results were performed by “ggplot2” packages (Ito & Murphy, 2013) in R 3.6.2.

## Result

### Identification of DEGs in PCa

**Figure 1:**
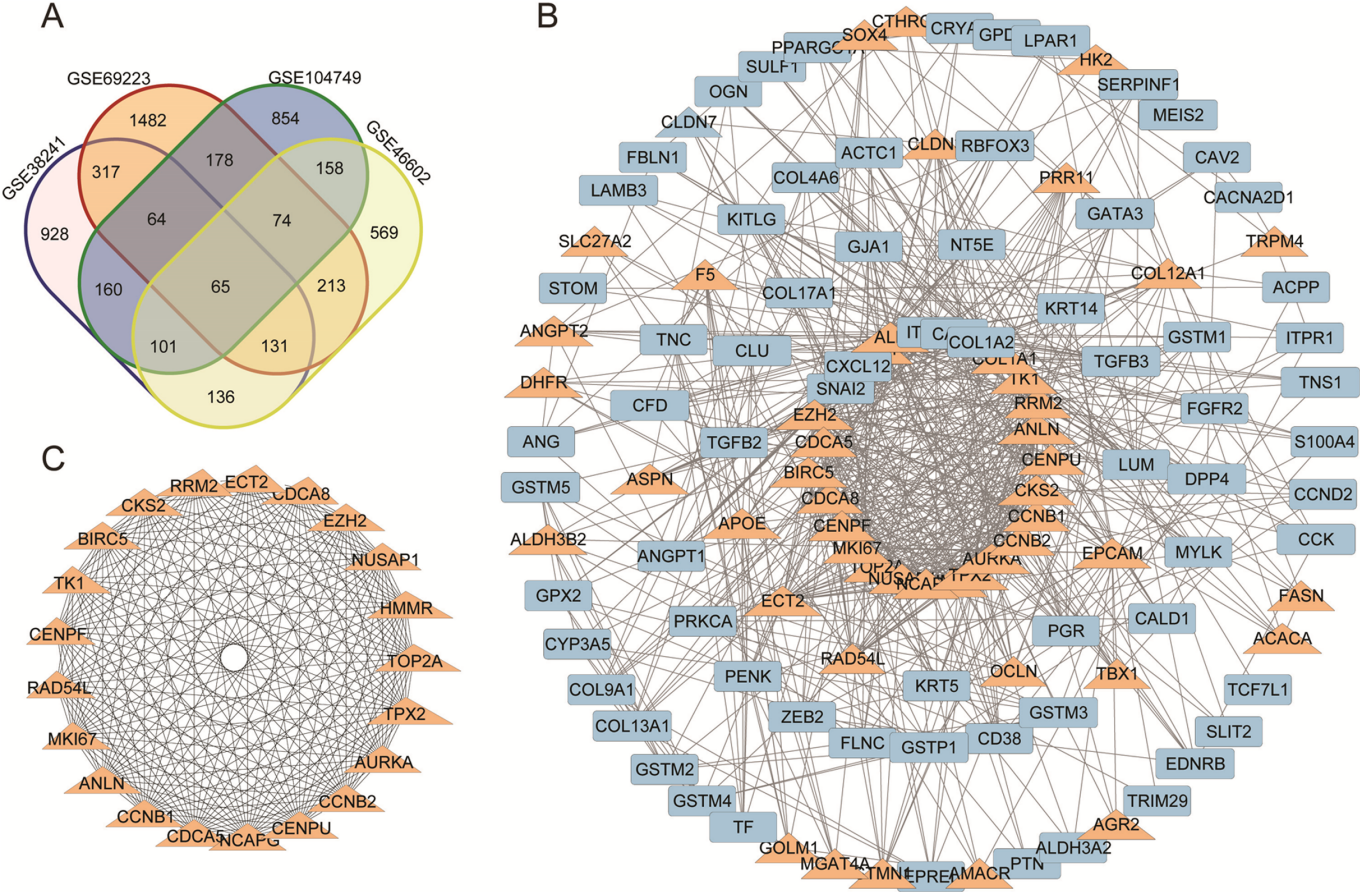
(A) DEGs Venn diagram of four prostate cancer microarray data. (B) PPI network for DEGs. (C) The most significant module of DEGs. (A) DEGs Venn diagram of GSE38241, GSE69223, GSE104749, GSE46602. As a result, 298 downregulated genes and 137 upregulated DEGs were screened, as shown in the Venn diagram. (B) The protein and protein interactions (PPI) 340 DEGs in 435 DEGs were performed in protein network and 167 DEGs were shown with degree >7. The upregulated genes were marked with orange,and the downregulated gene were marked with blue. (C) The most statistically significant module was acquired using Cytoscape.

A total of 298 downregulated genes and 137 upregulated DEGs were screened, as shown in the Venn diagram (Fig. 1A). Expression datasets GSE46602, GSE104749, GSE38241 and GSE69223 were obtained from the GEO database. GEO2R was used to screen the DEGs with the criteria of |log2FC| (fold change) ≥ 1 and *P*-value < 0.01. Afterward, we got 1,953 DEGs in GSE46602, 2,513 DEGs in GSE104749, 2,682 DEGs in GSE38241 and 2,639 DEGs in GSE69223. Considering the errors between the four datasets, we used the genes that met the screening criteria of any three datasets for the next analysis.

TCGA prostate cancer gene matrix was transformed by log2(exp + 1).

### KEGG and GO enrichment analyses of DEGs

DAVID was used to analyze the function classification of the 298 downregulated genes and 137 upregulated DEGs obtained by intersection. Gene Ontology results showed DEGs were significantly enriched in cell and biological adhesion, mesenchymal cell differentiation and the development of biological process. For the cellular component groups, the extracellular region and the matrix were significantly enriched. In the group analyzed for molecular function, the glutathione transferase activity, extracellular matrix structural constituent identical protein binding, and general identical protein binding were significantly enriched. KEGG pathway analysis revealed that the DEGs were mainly enriched in focal adhesion and in the glutathione metabolism pathway (Table 1).

**Table 1:** GO and KEGG pathway enrichment analysis of DEGs in prostate cancer samples. KEGG pathway analysis revealed that the DEGs were mainly enriched in focal adhesion and Glutathione metabolism pathway.

Pathway ID	Term	Count	*P*-value
GO:0007155	Cell adhesion	39	1.86E−06
GO:0007155	Biological adhesion	39	1.88E−06
GO:0048762	Mesenchymal cell differentiation	9	2.52E−05
GO:0014031	Mesenchymal cell development	9	2.52E−05
GO:0044421	Extracellular region part	63	3.08E−14
GO:0031012	Extracellular matrix	32	6.05E−11
GO:0005578	Proteinaceous extracellular matrix	29	9.94E−10
GO:0005578	Extracellular region	87	1.37E−09
GO:0004364	Glutathione transferase activity	6	7.31E−05
GO:0005201	Extracellular matrix structural constituent	10	1.57E−04
hsa04510	Focal adhesion	17	4.85E−05
hsa04510	Drug metabolism	9	1.64E−04
hsa04510	Glutathione metabolism	8	2.54E−04

**Note:**

GO, Gene Ontology; KEGG, Kyoto Encyclopedia of Genes and Genomes; DEGs, differentially expressed genes.

### PPI and module analysis

We acquired the PPI network and identified the most significant model based on the 298 downregulated genes and 137 upregulated DEGs. Cytoscape software and the online database STRING (available online: https://string-db.org/) were used to screen core genes. 340 DEGs in 435 DEGs were performed in protein network and 167 DEGs were shown with degree >7 (Fig. 1B). The most statistically significant module was acquired using Cytoscape (Fig. 1C). The KEGG and GO enrichment of this module were analyzed using DAVID. Results showed that the genes in the most significant module were mainly enriched in the functions of cell cycle phases, mitosis, cell division, the microtubule cytoskeleton, the p53 signaling pathway and Oocyte meiosis (Table 2).

**Table 2:** GO and KEGG pathway enrichment analysis of DEGs in the most significant module. The genes in the most significant module were mainly enriched in the function of cell cycle phase, mitosis, cell division, microtubule cytoskeleton, spindle, p53 signaling pathway, Oocyte meiosis.

Pathway ID	Term	Count	*P*-value
GO:0000279	M phase	14	6.27E−18
GO:0022403	Cell cycle phase	14	1.27E−16
GO:0007067	Mitosis	11	4.12E−14
GO:0000280	Nuclear division	11	4.12E−14
GO:0051301	Cell division	10	4.04E−11
GO:0015630	Microtubule cytoskeleton	9	1.90E−07
GO:0005819	Spindle	6	1.04E−06
GO:0051276	Chromosome organization	8	1.65E−06
GO:0044430	Cytoskeletal part	9	1.22E−05
GO:0003682	Chromatin binding	4	6.21E−04
hsa04115	p53 Signaling pathway	3	0.003543755
hsa04114	Oocyte meiosis	3	0.009071242

**Note:**

GO, Gene Ontology; KEGG, Kyoto Encyclopedia of Genes and Genomes; DEGs, differentially expressed genes.

### Candidate gene selection, analysis

**Figure 2:**
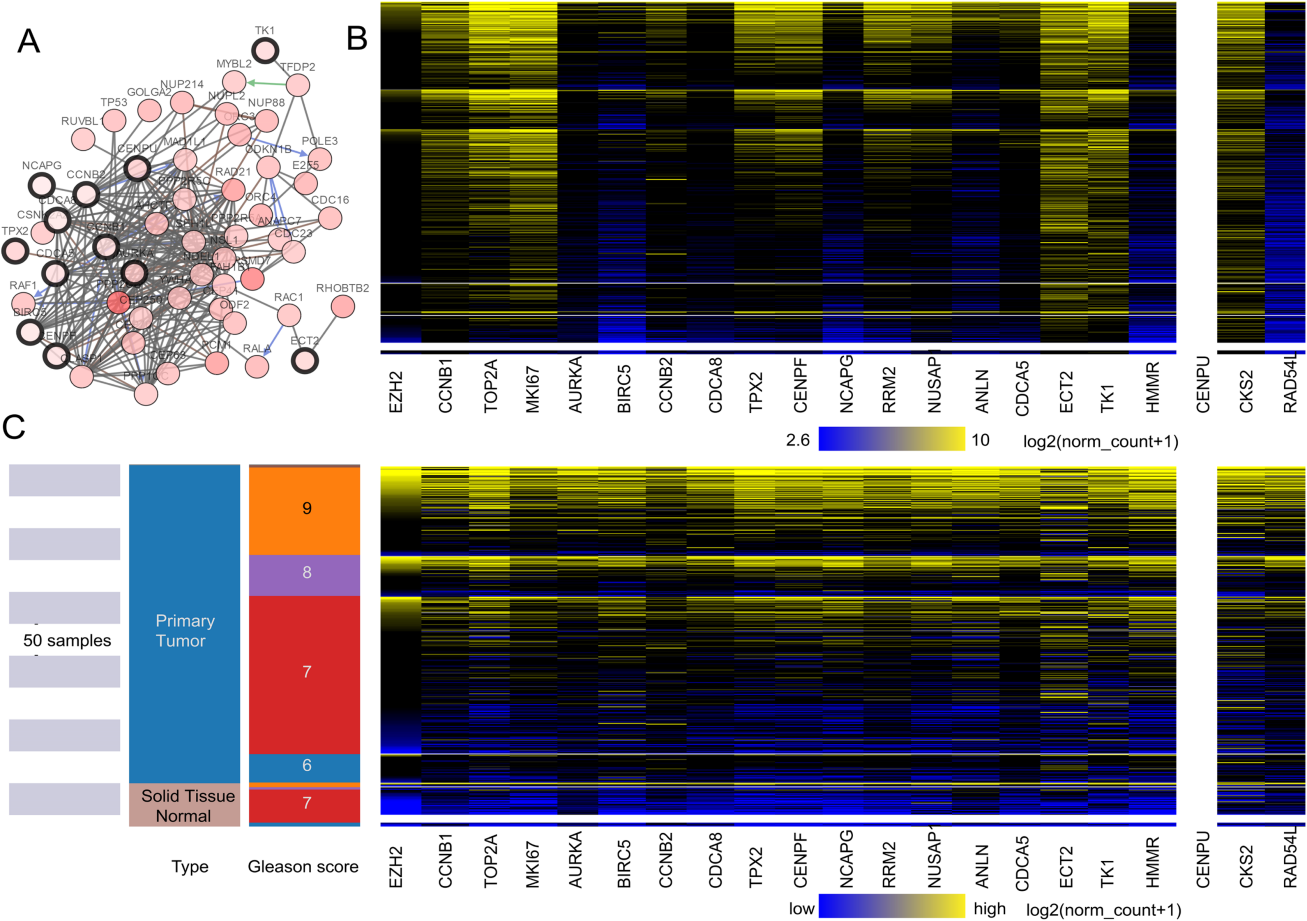
(A) The interaction network between the hub genes and their co-expression genes. (B and C) Heat map of hub genes by UCSC. (A) The interaction network between the hub genes and their co-expression genes by cBioPortal. Nodes with a black outline represent hub genes. Nodes with thin black genes represent co-expression genes. (B) The color parameters of C were adjusted as max color 100% saturation of log2(norm_count + 1) = 10.4; min color 100% saturation of log2(norm_count + 1) = 2.65. (C) Heat map of hub genes by UCSC. The samples of pink are normal tissue. The cancer samples are blue. High expressed genes were marked yellow. The low expressed genes were blue.

A total of 20 core genes with a degree ≥20 selected by MCODE were obtained from the protein–protein network, and they were considered to be candidate core genes. The co-expression network of core genes was analyzed by cBioPortal (Fig. 2A). Heatmap showed that the core genes in cancer groups upregulated more significantly than in normal groups by UCSC (https://xena.ucsc.edu) (Fig. 2C). In addition, the core genes were considered to have a close relation to the Gleason score (Fig. 2B). We adjusted the highest color according to 100% saturation parameters of log2 (norm_count + 1) ≥ 10.4 and the lowest color according to 100% saturation parameters of log2 (norm_count + 1) ≤ 2.65 (Fig. 2B). We compared solid normal tissue to primary tumor tissue (Figs. 2B and 2C). We found that CCNB1, TPX2, CENPF, TOP2A, MKI67, ECT2, TK1, RRM2, NUSAP1, CKS2 were overexpressed consistently in the 568 TCGA Prostate Cancer (PRAD) samples. Therefore, these genes were closely related to carcinogenesis and stage.

### Selection real core genes

**Figure 3:**
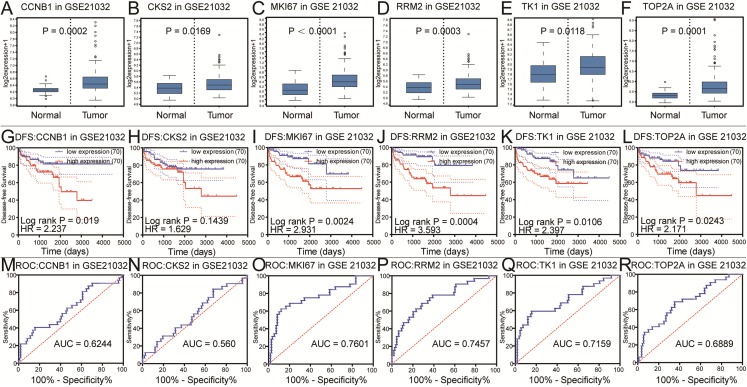
Differential analysis, disease-free survival analysis and ROC curve analysis in GSE21032. (A–F) The box plots of CCNB1, CKS2, MKI67, RRM2, TK1, TOP2A from GSE21032. The six core genes were found to be risk factors in GSE21032. (G–L) The disease-free survival KM curve of the six core genes were analysis by GSE21032, which showed prognosis value of prostate cancer patients. The Log-rank *P* value < 0.05 was considered to be significance. We recommended CKS2 as prognosis factors because the survival difference in 5 years is significance. (M–R) The ROC curve and AUC of the six core genes were showed.

**Figure 4:**
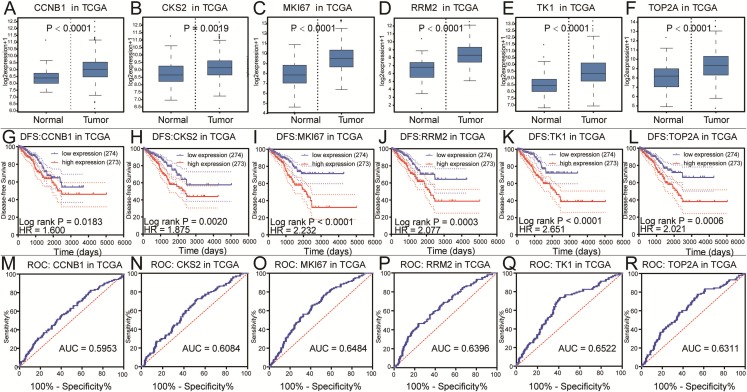
Differential analysis, disease-free survival analysis and ROC curve analysis in TCGA. (A–F) The box plots of CCNB1, CKS2, MKI67, RRM2, TK1, TOP2A from TCGA. The six core genes were identified to be risk factors in TCGA. (G–L) The disease-free survival KM curve of the six core genes were analysis by TCGA, which demonstrated prognosis value of prostate cancer patients. The Log-rank *P* value < 0.05 was considered to be significance. (M–R) The ROC curve and AUC of the six core genes were showed.

**Figure 5:**
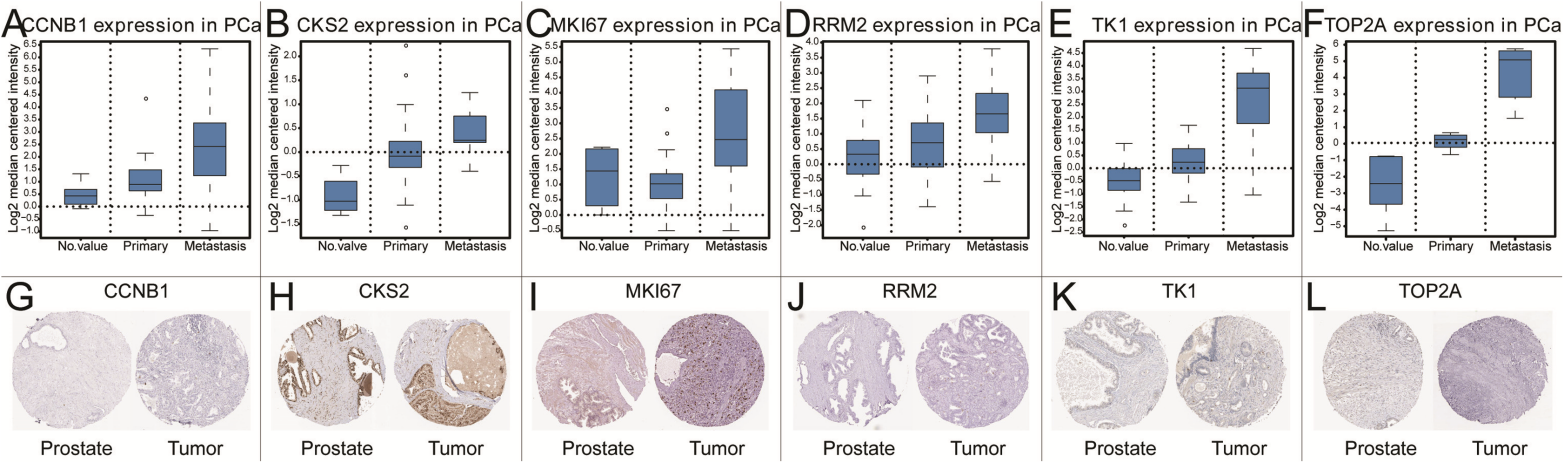
The gene expression of CCNB1, CKS2, MKI67, RRM2, TK1, TOP2A on transcriptional and translational level. Verification of the expression of core genes on transcriptional and translational level by Oncomine database and The Human Protein Atlas database (immunohistochemistry) CCNB1 (A and G), CKS2 (B and H), MKI67 (C and I), RRM2 (D and J), TK1 (E and K), TOP2A (F and L).

To further explore the relationship to phenotype and prognosis status, we performed differential analysis, disease-free survival analysis and ROC curve analysis in GSE21032 (Figs. 3A–3R). CCNB1, CKS2, RRM2, MKI67, TK1 and TOP2A were considered to be core genes with the lowest *P* value in the above 20 candidate genes. The results showed that MKI67 had the best prognostic value and the most significant *P* value (*P* = 0.0024). In addition, CCNB1, CKS2, RRM2, TK1 and TOP2A also showed closely clinical correlation. Based on the above analysis, the six genes were predicted to be the core factors affecting prostate cancer. To determine our prediction, we found CCNB1, CKS2, MKI67, RRM2, TK1 and TOP2A acted as independent prognostic factors in TCGA prostate cancer (Figs. 4A–4R). In order to verify the differential expression of key factors at the transcriptional and translational levels, we found cases of immunohistochemistry of core factors in the HPA database. In contrast to normal prostate tissue, we found that the cancer group stained deeper. At the same time, the expression difference of core genes was found between local lesions and metastatic patients in different external data sets of Oncomine database (Figs. 5A–5L). These findings clarified that core factors played an important role in the entire process of prostate cancer.

### Clinical relevance and GSEA

**Figure 6:**
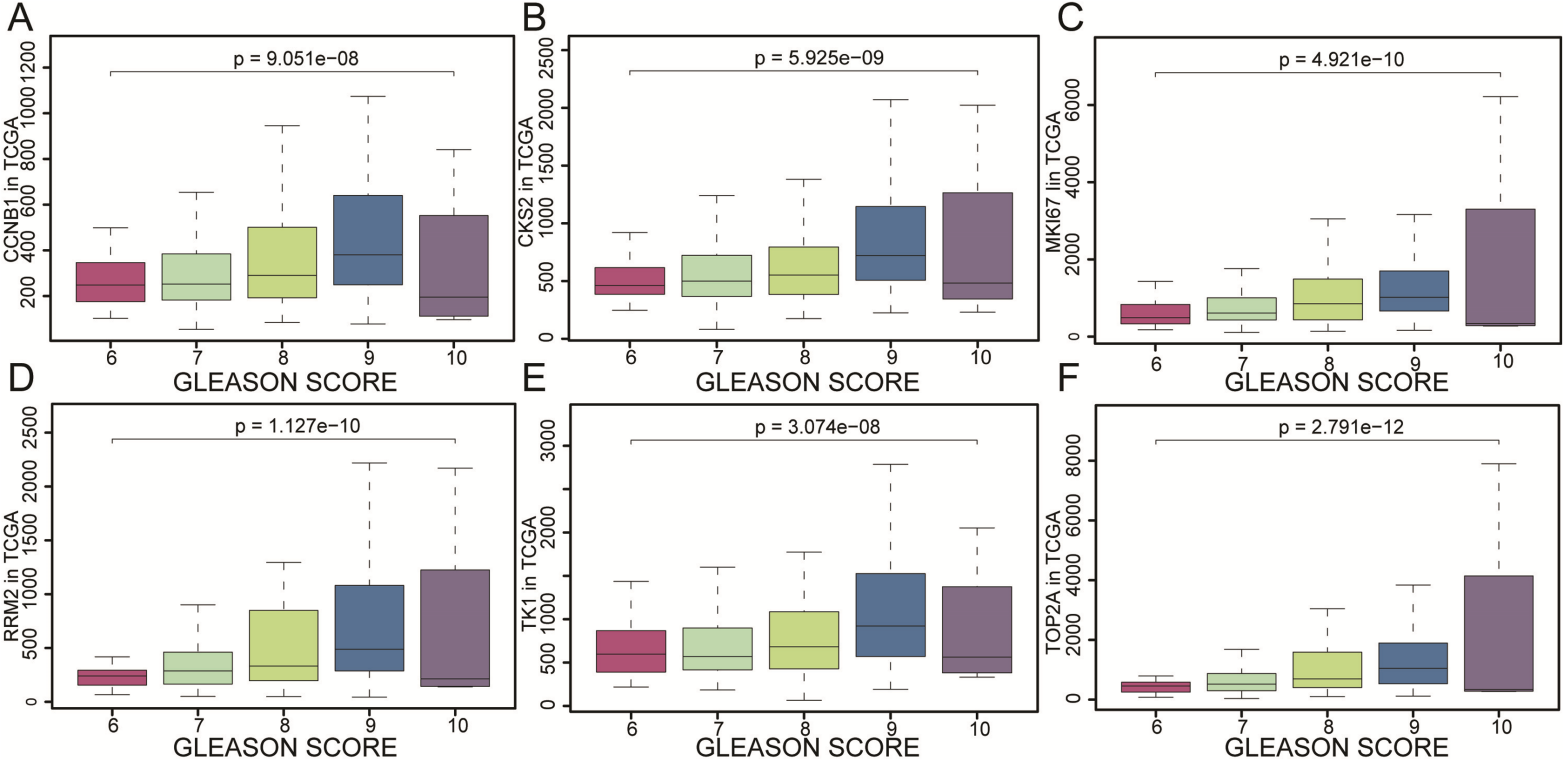
The gene expression of CCNB1, CKS2, MKI67, RRM2, TK1, TOP2A as represented by different Gleason scores. The gene expression of (A) CCNB1, (B) CKS2, (C) MKI67, (D) RRM2, (E) TK1, (F) TOP2A as represented by different Gleason scores. The results were evaluated by variance analysis.

**Figure 7:**
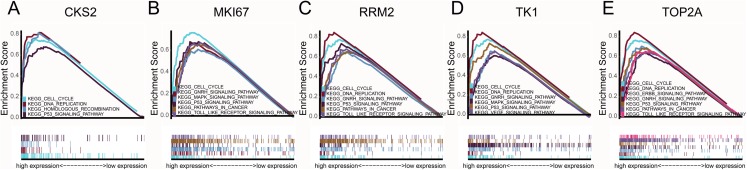
GSEA pathway enrichment analysis. GSEA pathway enrichment analysis of high expression groups of (A) CKS2, (B) MKI67, (C) RRM2, (D) TK1, (E) TOP2A. Pathway with FDR < 0.05 were considered to be significance.

CCNB1, CKS2, MKI67, RRM2, TK1 and TOP2A were found with positive clinical correlation to GLEASON SCORE (Figs. 6A–6F), and we could clearly identify the core factors as cancer risk factors, the expression level increased as Gleason score elevated. GSEA analysis suggested that cell cycle, DNA replication, GNRH signaling pathway, P53_signaling_pathway, were enriched in CKS2, MKI67, RRM2, TK1 and TOP2A high expression group jointly (Figs. 7A–7E). The *P*-value of core genes were listed (Table 3). Therefore, the core genes were positively related to the four cancer related pathways jointly.

**Table 3:** GSEA results of core genes in TCGA PRAD. The *P*-value of the P53_signaling_pathway, GNRH signaling pathway, DNA replication, cell cycle in the 5 core genes were listed.

	P53_signaling_pathway		GNRH_signaling_pathway		DNA_replication		Cell_cycle	
	NOM *P*-val	FDR *q*-val	NOM *P*-val	FDR *q*-val	NOM *P*-val	FDR *q*-val	NOM *P*-val	FDR *q*-val
RRM2	<0.001	0.0012	<0.001	4.65E−04	<0.001	0.001	<0.001	6.04E−04
CKS2	<0.001	0.0054	0.125	0.1249	<0.001	0.0059	<0.001	0.00254688
MKI67	<0.001	0.0026	<0.001	9.34E−04	<0.001	0.0023	<0.001	0.001120284
TK1	<0.001	0.0124	0.0653	0.0708	<0.001	0.0049	<0.001	0.003169282
TOP2A	0.001992032	0.0045	<0.001	0.0029	<0.001	0.004227	<0.001	0.00279397

## Discussion

Despite the advances that have been made in understanding the molecular processes at the onset and in the progression of this disease, PCa still remains high morbidity and mortality, particularly in low-income countries (Hernández et al., 2019). However, the formation mechanisms of PCa are incomprehensive. Therefore, we excavated potential biomarkers for diagnosis and treatment (Yang et al., 2018).

In the present study, 298 downregulated and 137 upregulated genes mainly enriched in cell adhesion, biological adhesion, regulation of cell proliferation and oxidation reduction. Epithelial cell adhesion molecule (Ep-CAM) is considered to have a critical role in carcinogenesis and cell proliferation (Tai et al., 2007). In addition, oxidoreductase activity often plays a crucial role in antioxidant defense and encodes tumor suppressors that are active in tumorigenesis (Li et al., 2017). Moreover, in the prostate carcinoma PC-3 cell model, the action of the gastrin releasing peptide (GRP) analog, bombesin (BN), on the activation of focal adhesion kinase (FAK) and its invasiveness suggests that this kinase might favor metastasis (Lacoste, Aprikian & Chevalier, 2005). In conclusion, these biological functions are closely related to the development and progression of prostate cancer.

Afterwards, a total of 20 candidate core genes were screened with degree ≥20. The full name and the function of the 20 core genes are listed (Table 4). CCNB1, CKS2, MKI67, RRM2, TK1, TOP2A were identified as real core genes by GSE21032 and validated in TCGA. The overexpression of CKS2 corresponds to metastasis and prognosis in various malignancies such as breast cancer, liver cancer and PCa (Yu, Zhong & Qiao, 2013). Other research showed that the forced expression of CKS2 has a positive correlation to cell growth, and also protects the cells from apoptosis (Lan et al., 2008). Thymidine kinase 1 (TK1) participates in DNA precursor synthesis and acts as a biomarker for malignant cancer including prostate and breast cancer (Jagarlamudi, Hansson & Eriksson, 2015). Meanwhile, research into serological TK1’s use in predicting precancer in a study involving 56,178 people showed that serological TK1 protein is a potential proliferative biomarker for early discovery of persons at risk for the development of, or who already have, malignancies or diseases associated with the development of malignancies (Wang et al., 2018). Moreover, TK1 is upregulated in the S phase of the cell cycle and its presence in cells is an indicator of active cell proliferation (Jagarlamudi & Shaw, 2018). The marker of proliferation, Ki-67 (MKI67), functions to mark tumor cell proliferation, including in the prostate, and has a close relation to the epithelial-mesenchymal transition (EMT) (Lindsay et al., 2016). Ki67 may improve the prediction of prostate cancer outcomes based on pathological standard parameters, improving prognosis as well as the monitoring of prostate cancer patients (Pascale et al., 2016). TOP2A is considered to be a biomarker for early identification of patients who have increased metastatic potential (Labbé et al., 2017). TOP2A encodes for topoisomerase IIα which controls the topology structure of DNA as well as cell cycle progression. This enzyme is a cell proliferation biomarker of cancer and normal tissue that is valuable for prostate cancer treatment (De Resende et al., 2013; Li et al., 2014). Cyclin B1 binds to CDC2 to ensure the transition toward mitosis by acting in the cell cycle from the G2 to M phase. High cyclin B1 levels, meanwhile, contribute to the development of polyploidy. Recent research has shown that Cyclin B1 is involved in breast, prostate cancer (Niranjan et al., 2016; Roh et al., 2005). A study also showed that elevated Cyclin B1 levels served as a biomarker for the prognosis of prostate cancer patients who were being treated with chemotherapy (Gomez et al., 2007). Ribonucleotide reductase regulatory subunit M2 (RRM2) is an enzyme that limits the rate of DNA synthesis and repair (Li et al., 2018; Wu et al., 2018). Mostly, RRM2 was overexpressed in PCa patients with a high Gleason score and an advanced T stage. RRM2 was considered to be a biomarker for prediction of recurrence in low risk PCa patients (Huang et al., 2014). In this study, these genes were confirmed to be the core genes in the same protein–protein network. The expression level performed consistently in prostate cancer. By the analysis of GSEA we found that these genes induced prostate cancer consistently by the cell cycle pathway and P53 signaling pathway and GNRH signaling pathway.

**Table 4:** Functional roles of 20 core genes with degree ≥20. These biological functions are closely related to the prostate cancer. A total of 20 core genes were screened with degree ≥20. The full name and the function of the 20 core genes were listed.

No.	Gene symbol	Full name	Function
1	CKS2	Cyclin kinase subunit 2	Overexpressed CKS2 promotes cell growth and protects cell from apoptosis
2	TK1	Thymidine kinase 1	TK1 is related to DNA precursor synthesis, acts as a proliferating biomarker of prostate and breast cancer
3	MKI67	Marker of proliferation Ki-67	KI-67 is a marker of tumor cell proliferation and the epithelial-mesenchymal transition
4	TOP2A	Topoisomerase IIα	TOP2A controls the topology structure of DNA and cell cycle progression
5	CCNB1	Cyclin B1	A high cyclin B1 level contributes to the development of polyploidy and was a prognosis biomarker of prostate cancer for chemotherapy
6	RRM2	Ribonucleotide-reductase regulatory subunit M2	RRM2 limits the rate of DNA synthesis and repair. It was considered to be a biomarker to predict recurrence in PCa patients with low-risk
7	EZH2	Enhancer of Zeste homolog 2	Drug targeting therapy EZH2 may be a new therapeutic strategy for advanced PCa and docetaxel-resistant PCa patients
8	AURKA	Aurora kinase A	AURKA was considered to be a potential prognostic biomarker for the progression of high-risk small-cell prostate cancer
9	BIRC5	Baculoviral IAP Repeat containing 5	BIRC5 may prevent apoptotic cell death, by survivin, a protein that inhibits apoptosis
10	CCNB2	Cyclin B2	CDC2 specifically binds to Cyclin B2 to increase cell migration which is related to the development of CRPC
11	CDCA8	Cell division cycle associated 8	Overexpressed CDCA8 is related to mitosis and cell growth and acts as a prognosis biomarker of breast cancer
12	TPX2	Targeting protein for Xenopus kinesin-like protein 2	TPX2 is a microtubule-associated protein linked to mitosis and spindle assembly and targeting TPX2 is a strategy of PCa
13	CENPF	Centromere protein F	CENPF acts in the centromere-kinetochore complex and chromosomal segregation, it is related to aggressive prostate cancer
14	NVAPG	Non-SMC condensin I complex subunit G	NCAPG acts as a target of miR-99a-3p in PCa cells, overexpression of NCAPG is related to CRPC
15	NUSAP1	Nucleolar and spindle-associated protein 1	NUSAP1 is a prognosis biomarker in the early stage of PCa patients
16	ANLN	Anillin actin binding protein	ANLN was found to be related to cell cycle and growth of PCa cells
17	CDCA5	Cell division cycle associated 5	Knockdown CDCA5 may cause cell cycle arrest in the G2/M phase
18	ECT2	Epithelial cell transforming sequence 2	ECT2 is a guanine nucleotide exchange factor that is related to the progression of tumors
19	HMMR	Hyaluronan-mediated motility receptor	Hyaluronan-mediated motility receptor (HMMR) binds native and fragmented HA, promotes HA uptake
20	CENPU	Centromere protein U	CENPU upregulation can increase the invasiveness of prostate cancer

Besides the six core genes we mentioned, we found EZH2, AURKA, BIRC5, CCNB2, TPX2, CENPF, NCAPG, NUSAP1 involved in the 20 candidate genes were demonstrated to be risk factors of prostate cancer. Zeste homolog 2 (EZH2) acts as the methyltransferase component of PRC2. Its enhancer regulation disorder is widely found in many aggressive, advanced cancers (Murashima et al., 2019). PRC2 inhibits stem cell self-renewal, cell cycle, cell differentiation and cell transformation through EZH2 (H3K27me3) modification (Alzrigat, Jernberg-Wiklund & Licht, 2018). Drug therapies targeting EZH2 may be a new strategy for advanced PCa and docetaxel-resistant PCa patients (Liu et al., 2019; Qiu et al., 2019). Aurora kinase A is encoded by the AURKA gene and has a vital function in the development of the cell cycle. It both controls and promotes entry into mitosis (López-Cortés et al., 2018). Studies have shown that AURKA is linked to pathological stage and distant metastasis in HCC (Chen et al., 2017). The AURKA gene has been proven to amplify in 67% of PCa patients with highly aggressive hormone-naive castration resistant cancer. AURKA, therefore, was considered to be a potential prognostic biomarker for the progression of high-risk small-cell prostate cancer’s resistance to castration (Park et al., 2014). One aspect of cancer is that apoptosis is uninhibited. Survivin, an inhibitor-of-apoptosis protein, is encoded by BIRC5, a gene that is linked to the regulation of apoptosis and cell division (Moore et al., 2014; Hmeljak et al., 2011). Studies have shown that BIRC5 is a biomarker for Oral Squamous Cell Carcinoma as well as breast, liver and prostate cancer. In PTEN deletion mouse model, there was a positive correlation between the survivin level and tumor growth. Researchers found that survivin plays an important role in the conversion process of prostatic intraepithelial neoplasia to adenocarcinoma (Adisetiyo et al., 2013) CCNB2 is linked to the process of transition from the G2 to the M phase. It has acted as a prognosis biomarker of non-small-cell lung cancer (Qian et al., 2015). Recently, studies have shown that CDC2 specifically binds to Cyclin B2 to increase cell migration, relating to development in CRPC (Huang et al., 2017; Manes et al., 2003). The targeting protein for Xenopus kinesin-like protein 2 (TPX2) is a microtubule associated protein that targets TPX2 repressed breast cancer by inhibiting the PI3k/AKT/P21 signaling pathway and activating the p53 pathway (Chen et al., 2018). High levels of chromosome missegregation is related to cell death and tumorigenesis. Recently, studies have shown that targeting TPX2 in breast and prostate cancer lowered the rate of chromosome missegregation, and have therefore regarded TPX2 as a candidate biomarker for treatment (Pan et al., 2017). Thus, TPX2 is considered to be a candidate target for PCa patients. Centromere protein F participates in cancer metabolism by regulating pyruvate kinase M2 phosphorylation signaling (Shahid et al., 2018). Recent work suggests that the upregulation of CENPF is linked to aggressive prostate cancer (Göbel et al., 2018). NCAPG has been demonstrated to be related to the overexpression of CCNB1. It is suggested to be a candidate target for HCC treatment (Zhang et al., 2018). The overexpression of Non-SMC condensing I complex subunit G (NCAPG) is involved in CRPC, and thus it may be a biomarker for PCa (Arai et al., 2018). Nucleolar and spindle-associated protein 1 (NUSAP1) is a prognosis biomarker in the in the earliest stage of PCa (Gordon et al., 2017). The overexpression of NUSAP1 may be related to the increased invasion and proliferation of PCa cells through the loss of RB1 (Gordon, Gulzar & Brooks, 2015).

Afterwards, we found the researches of CDCA8, ANLN, CDCA5, ECT2, HMMR, CENPU were poor, which the prognosis value and mechanisms needed further investigation. Cell division cycle associated 8 (CDCA8) over-expression is related to mitosis and tumor growth and may act as a prognosis biomarker in bladder cancer, cutaneous melanoma, breast cancer and osteosarcoma (Bi et al., 2018; Dai et al., 2015). The regulation mechanism of CDCA8 in PCa patients, however, is unclear and needed further study. ANLN was found to relate to the cell cycle and growth in PCa, but the regulation mechanisms are currently unknown (Takayama et al., 2019). CDCA5 knockdown led to cell cycle arrest in the G2/M phase (Tian et al., 2018). Epithelial cell transforming sequence 2 (ECT2) acts as an exchange factor of the guanine nucleotide, which is related to the progression of cell division regulation and the cell cycle (Bai et al., 2018). Hyaluronan-mediated motility receptor (HMMR) promotes HA uptake, and related time to biochemical failure in Gleason score 7 tumor (Rizzardi et al., 2014). Overexpression of CENPU is related to breast cancer, lung cancer, ovarian cancer and prostate cancer. Upregulated CENPU can increase the invasiveness of PCa cells (Winter et al., 2017).

In this study, we identified CKS2, TK1, MKI67, TOP2A, CCNB1 and RRM2 as crucial components in the diagnosis and treatment of PCa. They acted together in the same pathway to induce prostate cancer. The research of the correlation between molecules in cell cycle, DNA replication, GNRH signaling pathway, P53_signaling pathway could reveal the underlying causes of cancer and provide novel ideas for research into target drugs. Meanwhile, the expression difference of CDCA8, ANLN, CDCA5, ECT2, HMMR and CENPU were significantly which needed in-deep study.

## Conclusion

This study excavated the core genes of prostate cancer, analyzed their functions, pathways, and their phenotype by means of reliable bioinformatics analysis of multiple datasets. The core genes in this study were considered to be potential targets and biomarkers, providing new ideas for the diagnosis and treatment of prostate cancer. More experimental studies are needed, however, to verify the mechanisms of these genes in prostate cancer.

## Additional Information and Declarations

### Data Availability

The following information was supplied regarding data availability: Data is available at NCBI GEO: GSE38241, GSE69223, GSE46602, GSE104749.
